# Effects of Immunocytokine Combined with Cattle Encephalon Glycoside and Ignotin on CTGF, HO-1 and NT-3 in Patients with Type 2 Diabetic Peripheral Neuropathy

**Published:** 2017-12

**Authors:** Jianguo SUN, Hui ZHENG, Xiuxia QIN, Liqin QI

**Affiliations:** 1. People’s Hospital of Rizhaolanshan, Rizhao, Shandong, PR China; 2. People’s Hospital of Wulian, Rizhao, Shandong, PR China

**Keywords:** Immune cytokines, Cattle encephalon glycoside and ignotin, Diabetic peripheral neuropathy

## Abstract

**Background:**

This study was designed to explore the correlation of connective tissue growth factor (CTGF), heme oxygenase (HO-1), neurotrophic factors (NT-3) with type 2 diabetic peripheral neuropathy, as well as the changes after immune cytokine alone and combined with cattleencephalon glycoside and ignotin treatment.

**Methods:**

Seventy-six patients with type 2 diabetes and peripheral neuropathy charged into People’s Hospital of Rizhaolanshan, China from 2014–2016 were selected. The severity of neuropathy was evaluated by TCSS. Pearson analysis was used to analyze the correlation between the degree of neuropathy and CTGF, HO-1 and NT-3. The patients were randomly divided into control group and observation group, n=38. The control group accepted TGF-β1 treatment on the basis of controlling diet and blood sugar, while the observation group was treated with cattle encephalon glycoside and ignotin injection on the basis of control group. CTGF, HO-1, NT-3 concentration in the blood and nerve conductive velocity (NCV) were detected and analyzed before and after treatment.

**Results:**

CTGF(r=−0.865), HO-1(r=−0.706), NT-3(r=−0.587) was negatively correlated with TCSS scores. After treatment, the concentrations of CTGF, HO-1and NT-3 in the observation group were higher than the control group (*P*<0.05). In moderate and severe lesions, the concentrations of CTGF, HO-1and NT-3 in the observation group were higher than the control group (*P*<0.05). The conduction velocity of nerve increased with the increase of CTGF, HO-1 and NT-3 concentrations. The obvious effective rate and total effective rate of observation group were both higher than the control group.

**Conclusion:**

Immune cytokine TGF-β1 combined with cattle encephalon glycoside and ignotin injection could improve the contents of CTGF, HO-1 and NT-3, and be better to treat the peripheral neuropathy of type 2 diabetes.

## Introduction

Type 2 diabetes is a multiple chronic metabolic disease. It results from the relative lack of insulin or the weak ability of insulin to degrade blood sugar; it is an invisible killer threatening the life of elderly people. It has the characteristics of complex factors and difficulty for healing (1).

At present, with the irregular lifestyle and unreasonable diet, most type 2 diabetes patients have a higher BMI, and with a variety of complications. Hundreds of diabetes complications have been confirmed, the harm to the body is astonishing (2). Diabetic peripheral neuropathy occurs in the microvascular, with a variety of clinical manifestations, strong latency, difficulty to discover at early stage and high incidence. The main manifestations are nerve-related sensory and motor function damage, unresponsive or too sensitive (3, 4). At present, the mechanism of peripheral neuropathy caused by diabetes is not very clear, the treatment is also difficult, in which the inflammatory effect is an important factor (5, 6). Some immune cytokines can reduce neuronal apoptosis and protect neuronal cells, while cattle encephalon glycoside and ignotin can promote the growth, differentiation and regeneration of neurons. Connective tissue growth factor (CTGF) is associated with the formation of connective tissue, which can indirectly affect nerve cells. The anti-inflammatory and anti-oxidative functions of heme oxygenase (HO-1) are important indicators of the degree of disease. Neurotrophic factor (NT-3) and nerve conduction velocity (NCV) can be the most intuitive indicator of the state of the nerve.

In this study, immune cytokines and cattle encephalon glycoside and ignotin were used to treat patients with type 2 diabetic peripheral neuropathy, and the effects of CTGF, HO-1 and NT-3 were analyzed in order to provide the basis for the pathogenesis and clinical treatment.

## Materials and Methods

### General Information

Patients with type 2 diabetes with peripheral neuropathy charged into People’s Hospital of Rizhaolanshan from July 2014 to July 2016 were selected. The inclusion criteria were in reference to the type 2 diabetes with peripheral neuropathy diagnostic criteria from WHO. The definition of the type 2 diabetes from ADA in 2010 (7):Glycosylated hemoglobin HbA1c≥6.5%; Fasting blood-glucose FPG≥7.0 mmol/L. Fasting is defined as at least 8 hours without calorie intake; The oral glucose tolerance test 2 hours blood glucose ≥11.1mmol/L; patients with typical high blood sugar or high blood sugar crisis symptoms, random blood glucose≥11.1mmol/L.The definition of the type 2 diabetic nephropathy (8) :UAE20 ∼ 200 μg/min, microalbuminuria period, clinical diagnosis of early diabetic nephropathy, while UAE continues over 200 g/min or conventional urine protein continues over 0.5 g/24h, which is the clinical diagnosis of diabetic nephropathy.

Exclusion criteria: 1) peripheral neuropathy patients caused by other factors than diabetes; 2) liver, brain, kidney and other vital organs function severely damaged; 3) neuroma and cancer patients. There were 76 patients with type 2 diabetes with peripheral neuropathy, 40 males and 36 females, aged (33–79) yr, mean age (57.1 ± 22.1) yr, duration of diabetes (6 to 26) yr, average (14.1 ± 6.9) yr, the course of diabetic peripheral neuropathy was (0.5 ∼ 5.8) yr, the average (3.2 ± 2.4) yr. According to the different treatment plan, patients were divided into the control group and observation group, the information can be seen from Table 1, there was no significant difference in terms of basic data between the two groups (*P*>0.05). This study was approved by the Ethics Committee and informed the patient to sign the informed consent.

**Table 1:** Basic Data (n, x̄±s)

***Group***	***Number of cases***		***Age (yr)***	***TCSS Score***			***Duration of Diabetes (year)***	***Duration of peripheral neuropathy (year)***	***Fasting blood sugar(mmol/L)***
	**Male**	**Female**		**6–8**	**9–11**	**12–19**			
Control	21	17	56.8±21.6	9	18	11	13.9±6.7	3.3±2.3	8.1±0.7
Observation	20	18	57.5±20.4	10	19	9	14.4±6.9	3.2±2.4	8.3±0.9
t/χ^2^	0.147		10.231	0.719			11.541	14.186	9.989

### Treatment

The control group was treated with immune cytokines TGF-β1 (PeproTech, USA) on the basis of routine therapy such as diet, blood glucose control. Intravenous infusion of sodium chloride injection with 5μgTGF-β1 was given once every 2 days, 3 weeks was a course of treatment. On the basis of the control group, the observation group was given intravenous infusion of sodium chloride injection containing 8ml cattle encephalon glycoside and ignotin (Jilin Si Chang Pharmaceutical Co., Ltd.) once a day, 3 weeks as a course of treatment.

The levels of CTGF, HO-1 and NT-3 in the blood of patients before and after treatment were measured with Elisa kit (Abcam, USA). The velocity of the median nerve and peroneal nerve in the motor nerve and sensory nerves was measured using the American Noraxon TeleMyo electromyography.

### Criteria

#### TCSS Scoring Criteria (7)

Degree of neuropathy was evaluated by TCSS scoring system, mainly by the neurological symptoms, nerve reflex, body sensory 3 most of the scores, detailed evaluation of the situation was in Table 2, normal: 0 to 6 points, 0 cases; mild: 6 to 8 points, 19 cases; moderate: 9 to 11 points, 37 cases; severe: more than 12 points, 20 cases.

**Table 2:** Distribution of TCSS Score

***Item***	***Index***	***Score criteria***
Neurological symptoms	Burning in the lower limbs, numbness, imbalance walking, fatigue, needle-like sharp pain, upper extremity with similar symptoms (6 items)	1 point for each indicator, a total of 6 points
Nerve reflex	Bilateral knee reflex, ankle reflex (4 items)	2 point for each indicator,1 point for weakened, a total of 8 points
Body feel	Foot toe haptic perception, temperature sensation, pain, vibration sense, sense of the position (5 items)	1 point for each indicator, a total of 5 points

#### Criteria for efficacy (8)

1) Markedly effective: clinical symptoms disappeared, normal tendon reflex, electromyogram nerve conduction velocity (NCV) increased> 5 mv/s or returned to normal; 2) effective: clinical symptoms reduced, tendon reflex improved, electromyogram NCV increased<5 mv / s; 3) invalid: no improvement in clinical symptoms, no improvement in tendon reflex, no change in electromyogram NCV.

### Statistical analysis

All data were processed using SPSS 16.0 statistical software (Beckham Information Technology Co., Ltd.). Factor correlation analysis used Pearson analysis, measurement data were expressed as x̄±s, the mean comparison used t test and single factor analysis of variance, enumeration data used χ2 test, *P* <0.05 for the difference was statistically significant.

## Results

**Fig. 1:**
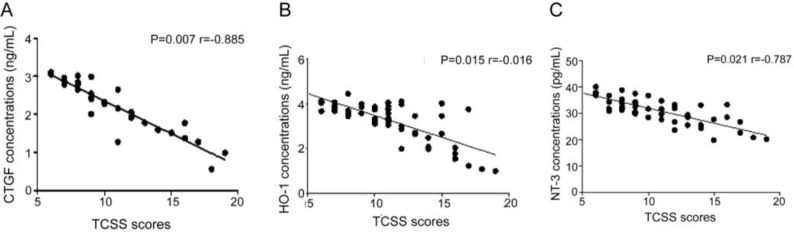
Correlation between TCSS score and CTGF, HO-1, NT-3 concentration. Figure 1A–C shows the correlation between CTGF, HO-1 and NT-3 concentrations and TCSS scores, respectively. The results showed that the concentration of CTGF, HO-1 and NT-3 was negatively correlated with TCSS score

The concentration of CTGF, HO-1 and NT-3 was negatively correlated with TCSS score. The *P* values were 0.007, 0.015 and 0.021 respectively, and the correlation coefficients were −0.865, −0.706 and −0.787 (Fig. 1).

**Fig. 2:**
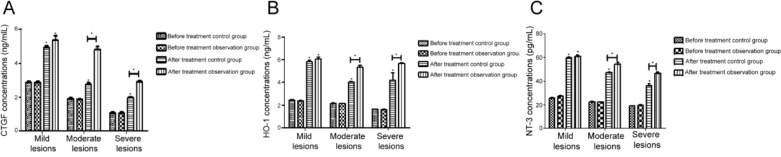
The effect of two kinds of treatments on the levels of CTGF, HO-1 and NT-3 were observed in the treatment of three levels of lesions. In the treatment of moderate and severe lesions, the levels of CTGF, HO-1 and NT-3 in the observation group were higher than those of the control group (*P*<0.05). 2A was CTGF concentration, 2B was HO-1 concentration, and 2C was NT-3 concentration.

After treatment, the levels of CTGF, HO-1 and NT-3 in both groups were higher than those before the treatment (*P* <0.05), and the levels of CTGF, HO-1 and NT-3 in the observation group were higher than those in the control group (*P* <0.05) (Table 3). There was no significant difference in the three protein factors between the observation group and the control group for mild lesions. The levels of CTGF, HO-1 and NT-3 in the observation group were higher than those in the control group for moderate and severe lesions (*P* <0.05) (Fig. 2).

**Table 3:** Changes in CTGF, HO-1, NT-3 concentrations before and after treatment (x̄±s)

***Group***	***Number of cases***	***Stage***	***CTGF (ng/mL)***	***HO-1 (ng/mL)***	***NT-3 (pg/Ml)***
Control	38	Before treatment	1.95±0.78	2.11±0.34	19.45±2.22
		After treatment	3.45±1.67^*^	4.05±0.56^*^	38.12±4.32^*^
Observation	38	Before treatment	1.91±0.69	2.08±0.31	20.12±2.03
		After treatment	5.03±1.02^*^#	6.12±0.21^*^#	59.32±5.01^*^#

* Indicates that compared with before treatment ^*^ *P*<0.05,

# Indicates that compared with the control group # *P*<0.05

The conduction velocity of nerve increased with the increase of CTGF, HO-1 and NT-3 concentration (*P* <0.05) (Table 4).

**Table 4:** NCV at different CTGF, HO-1, NT-3 levels (m/s, x̄±s)

***Indicator***	***Concentration range***	***Number of cases***	***Motor nerve***		***Sensory nerve***	
			**Median nerve**	**Peroneal nerve**	**Median nerve**	**Peroneal nerve**
CTGF (ng/mL)	Low (<1.5)	18	46.12±4.67	41.76±2.02	43.21±5.01	42.03±2.11
	Medium (1.5–4)	38	49.87±4.23^*^	45.11±4.43^*^	47.87±4.87^*^	48.54±4.32^*^
	High (>4)	20	54.23±2.78^*^#	51.22±3.67^*^#	52.01±4.21^*^#	55.12±5.23^*^#
HO-1 (ng/mL)	Low (<2.1)	19	45.32±4.11	39.21±2.65	42.33±4.77	41.43±3.54
	Medium (2.1–4.5)	37	50.11±4.32^*^	43.33±4.67^*^	47.22±3.54^*^	48.89±4.33^*^
	High (>4.5)	20	56.43±4.23^*^#	53.22±2.11^*^#	51.33±3.87^*^#	55.54±5.09^*^#
NT-3 (pg/mL)	Low (<30)	17	43.23±3.25	38.22±2.12	40.11±2.43	41.09±3.22
	Medium (30–50)	35	50.21±4.56^*^	46.32±3.43^*^	47.22±4.12^*^	49.22±4.01^*^
	High (>50)	24	59.23±4.21^*^#	55.23±3.33^*^#	53.21±2.54^*^#	56.21±3.43^*^#

* Indicates that compared with low level, **p**<0.05;

# Indicates that compared with medium level, **p**<0.05.

The effective rate and the total effective rate in the observation group was significantly higher than the control group, and the difference was statistically significant (*P* <0.05) (Table 5).

**Table 5:** Comparison of treatment efficacy in two groups (n (%))

***Group***	***Number of cases***	***Marked effective***	***Effective***	***Total effective rate(%)***
Control	38	13 (34.2)	20 (52.6)	86.8
Observation	38	6 (15.8)	18 (47.4)	63.2
χ^2^	——	9.028	0.541	14.852
P	——	0.003	0.462	0

## Discussion

Type 2 diabetes is a metabolic disease prevalent around the world, due to high concentrations of blood glucose damaging the body’s normal internal environment, disrupting the signal transmission between the signaling pathway, resulting in a high opportunity of illness to other organs (8, 9). Peripheral nerve damage of type 2 diabetes patients can be caused by hyperglycemia, dyslipidemia, metabolic inflammation, leading to numbness of the limbs, abnormal reactions; some protein factor concentration in the blood may also change (10).

Immune cytokines are active proteins secreted into the blood by immune cells such as T cells, NK cells, B cells, etc., in which several have been discovered and capable of mass production (11). Among them, TGF-β1 (transforming growth factor-β1) has the role of inhibition of inflammation and neuronal apoptosis, and promoting the expression of nerve-related factors (12). TGF-β1 has a certain role in promoting the survival of facial nucleus motor neurons (13). Cattle encephalon glycoside and ignotinis a complex biological agent consisting of a variety of active protein factors, gangliosides and nucleic acids that play a positive role in the prevention and repair of diseased nerve cells and tissues. It can also promote stem cells differentiation to neural stem cells, and mature neuronal cell further, repairing defective nerve tissue (14–16). The results of this study also confirmed that the treatment efficiency in the observation group of cattle encephalon glycoside and ignotin combined with TGF-β1 was significantly higher than that of TGF-β1 alone. Diabetic patients with neuropathy are able to stimulate high levels of TGF-β1, and TGF-β1 level of serum is relatively higher (17).

Part of the protein factors in the blood has certain relevance and an indicating function on nerve function changes (17, 18). CTGF can keep the cells in normal amplification and movement, playing an important role in the fibrosis of nerve tissue. Maeda N and others confirmed that TGF-β can increase the expression of CTDF in skeletal muscle cells of L6 mice (19). HO-1 can delay the aging of tissues and organs, eliminate inflammation in the body. NT-3 can promote the survival, growth and differentiation of neurons, and stimulate the formation of neurite outgrowth (19, 20). NCV is positively correlated with the severity of neurological disorders. The slower the NCV is, the more severe the neuropathy is, the worse the ability of the human body to perceive the surrounding stimuli. Controlling blood sugar can reduce fall of NCV, but cannot completely eliminate (20).

In this study, CTGF, HO-1 and NT-3 were highly correlated with type 2 diabetic peripheral neuropathy, CTGF, HO-1 and NT-3 were negatively correlated with TCSS scores, and the correlation coefficients were −0.865, −0.706, −0.587 (*P*<0.05). In the moderate and severe lesions, the levels of CTGF, HO-1 and NT-3 in the observation group were higher than those in the control group. The conduction velocity of nerve increased with the increase of CTGF, HO-1 and NT-3. The above three kinds of protein factors changed towards concentration of normal neurological function after the treatment, which indirectly illustrates the effect of treatment, but the relevant mechanisms of elevation and the process still need further exploration.

## Conclusion

The levels of CTGF, HO-1 and NT-3 could better reflect the degree of neuropathy. Immune cytokine TGF-β1 combined with cattle encephalon glycoside and ignotin could increase the levels of CTGF, HO-1 and NT-3, which has a better treatment effect for treating type 2 diabetic peripheral neuropathy.

## Ethical considerations

Ethical issues (Including plagiarism, informed consent, misconduct, data fabrication and/or falsification, double publication and/or submission, redundancy, etc.) have been completely observed by the authors.
